# Age-Related Differences in Clinical and Psychosocial Predictors of Unmet Needs in Bladder Cancer Survivors

**DOI:** 10.1093/geroni/igab046.1096

**Published:** 2021-12-17

**Authors:** Nihal Mohamed, Tung Ming Leung, Katherine Ornstein, Naomi Alpert, Travonia Brown-Hughes, Emanuela Taioli, Natasha Kyprianou

**Affiliations:** 1 Icahn School of Medicine at Mount Sinai Department of Oncological Sciences, New York, New York, United States; 2 Northwell Health, Northwell Health, New York, United States; 3 Icahn School of Medicine at Mount Sinai, New York, New York, United States; 4 School of Pharmacy, Hampton, Virginia, United States

## Abstract

Understanding of unmet needs and their predictors among bladder cancer (BC) survivors is critical to optimize health care planning for patients. This study compares between younger (<65 Years) and older (≥65 Years) BC patients across seven domains of unmet needs (e.g., informational, psychological, supportive care, daily living, communication, logistic, and sexuality needs) and their demographic, clinical, and psychosocial predictors. BC survivors (N=159; 47% women) were recruited from the Bladder Cancer Advocacy Network and completed a questionnaire that included the needs assessment survey (BCNAS-32), hospital anxiety and depression scale (HADS), coping (BRIEF COPE), social provisions scale (SPS), and self-efficacy beliefs (GSE) scale. Although no significant group differences in all reported needs emerged, both groups reported more communication (IQR = 50 (62.5) and less sexuality needs (IQR =13 (52.1). Older patients reported higher depression and anxiety (IQR = 32 (11.5); N = 68) than younger patients (IQR = 28 (11.0); p < .01; N = 88). Multivariable analyses stratified by age showed significant effects of gender among older patients with women experiencing more psychological, care, communication, and sexuality needs than men. Multivariable analyses also showed age-related differences (p < .05) in the predictors of needs controlling for covariates (e.g., gender). Among older patients both higher depression and anxiety and lower self-efficacy beliefs were associated with more psychological, care, and communication needs. Among younger patients, higher depression and anxiety were associated with more psychological, logistic, daily living, and communication needs. Results emphasize the importance of tailoring care planning for patients based on age.

